# Changes of Erythrocyte Fatty Acids after Supplementation with Highly Concentrated Docosahexaenoic Acid (DHA) in Pediatric Cystic Fibrosis: A Randomized Double-Blind Controlled Trial

**DOI:** 10.3390/jcm12113704

**Published:** 2023-05-26

**Authors:** Roser Ayats-Vidal, Montserrat Bosque-García, Begoña Cordobilla, Oscar Asensio-De la Cruz, Miguel García-González, Jesús Castro-Marrero, Irene López-Rico, Joan Carles Domingo

**Affiliations:** 1 Pediatric Allergies, Immunology and Pneumology Unit, Pediatric Medicine Service, Institut d’Investigació i Innovació Parc Taulí (I3PT-CERCA), Parc Taulí Hospital Universitari, Universitat Autònoma de Barcelona, Parc Taulí 1, E-08208 Sabadell, Spain; mbosque@tauli.cat (M.B.-G.); oasensio@tauli.cat (O.A.-D.l.C.); mgarciago@tauli.cat (M.G.-G.); 2 Department of Biochemistry and Molecular Biomedicine, Faculty of Biology, University of Barcelona, E-08028 Barcelona, Spain; bgcordobilla07@ub.edu; 3 ME/CFS Research Unit, Division of Rheumatology, Vall d’Hebron Research Institute, Universitat Autònoma de Barcelona, E-08035 Barcelona, Spain; jesus.castro@vhir.org; 4 Pharmacy Department, Institut d’Investigació I Innovació Parc Taulí (I3PT-CERCA), Parc Taulí Hospital Universitari, Universitat Autònoma de Barcelona, E-08208 Sabadell, Spain; ilopezr@tauli.cat

**Keywords:** cystic fibrosis, fatty acids, docosahexaenoic acid, eicosapentaenoic acid, omega-3, pancreatic insufficiency, randomized controlled study

## Abstract

We characterized the fatty acid profiles in the erythrocyte membrane of pediatric patients with cystic fibrosis (CF) receiving highly concentrated docosahexaenoic acid (DHA) supplementation (Tridocosahexanoin-AOX^®^ 70%) at 50 mg/kg/day (*n* = 11) or matching placebo (*n* = 11) for 12 months. The mean age was 11.7 years. The DHA group showed a statistically significant improvement in n-3 polyunsaturated fatty acids (PUFAs), which was observed as early as 6 months and further increased at 12 months. Among the n-3 PUFAs, there was a significant increase in DHA and eicosapentaenoic acid (EPA). Additionally, a statistically significant decrease in n-6 PUFAs was found, primarily due to a decrease in arachidonic acid (AA) levels and elongase 5 activity. However, we did not observe any changes in linoleic acid levels. The long-term administration of DHA over one year was safe and well tolerated. In summary, the administration of a high-rich DHA supplement at a dose of 50 mg/kg/day for one year can correct erythrocyte AA/DHA imbalance and reduce fatty acid inflammatory markers. However, it is important to note that essential fatty acid alterations cannot be fully normalized with this treatment. These data provide timely information of essential fatty acid profile for future comparative research.

## 1. Introduction

Cystic fibrosis (CF) is an autosomal recessive disease considered to affect at least 100,000 people worldwide [1]. The disease is caused by mutations in the cystic fibrosis transmembrane conductance regulator (*CFTR*) gene coding for the *CFTR* protein, which is expressed at the surface of epithelial cells. The *CFTR* protein functions predominantly as a chloride and bicarbonate channel, although it has roles in transporting other ions and modifying the function of other ion transporters [2]. Dysfunction of the *CFTR* protein causes the accumulation of exocrine secretions in multiple systems, with marked mucus hyperproduction and plugging especially in the smallest airways and the pancreatic ducts. Respiratory, gastrointestinal, and pancreatic manifestations are prominent clinical manifestations in patients with CF, although lung disease still remains the main reason for the high morbidity and mortality of the disease [3].

CF patients typically present with abnormal essential fatty acid profiles in lipid membranes, characterized by low levels of docosahexaenoic acid (DHA) (C22:6 n3) and linoleic acid (LA) (C18:2 n6) and increased levels of ARA/DHA ratio [4,5,6,7], with all of these contributing to the exaggerated inflammatory response in CF. The imbalance between omega-3 long-chain polyunsaturated fatty acids (n-3 PUFAs) and omega-6 PUFAs (n-6 PUFAS) in cell membranes is consistent with overall suppression of the anti-inflammatory pathway mediated by n-3 PUFAs metabolites and exacerbation of the inflammatory pathway mediated by metabolites of ARA [8]. Further, in the serum of people with CF, there are higher dihomo-γ-linolenic acid (DHGLA), oleic acid (OA), and mead acid serum concentrations [9]. 

Although some authors initially referred to these alterations in the fatty acid profile only being present in CF patients with pancreatic insufficiency [10,11], other, later studies have shown that CF patients with pancreatic sufficiency also present some alterations in the FA profile, such as long-chain non-esterified fatty acid patterns [11,12].

However, despite extensive investigations, the underlying mechanisms for fatty acid alterations in CF and the connecting link to *CFTR* protein expression dysfunction are still poorly understood [13,14,15]. Some of the theories that support these conclusions are increased turnover in cell membranes [16], a defect in desaturase activity [17], increased fatty acid oxidation (lipid peroxidation) [18], and an increase in the production of eicosanoids [19,20].

New insights into the role of the metabolism of fatty acids, highlighted by findings in experimental studies showing that symptoms and biochemical aberrations of CF could be normalized by high doses of DHA [6,21,22,23], have been the rationale for therapeutic strategies based on omega-3 fatty acid supplementation with DHA and eicosapentaenoic acid (EPA) to patients with CF. Although numerous clinical studies of supplementation with omega-3 fatty acids in CF have been reported [24,25,26,27,28,29,30], results regarding the improvement of clinical parameters and benefits on inflammatory biomarkers are inconsistent [31]. The methodological heterogeneity of the published studies is explained by notable differences in the epidemiological design (randomized controlled trials, longitudinal studies), duration of supplementation, use of variable concentrations of omega-3 fatty acids—especially DHA (e.g., algal source or derived from fish oils), and selection of the variables of interest. In a recent Cochrane systematic review of five randomized controlled trials with 106 participants (children and adults) undergoing omega-3 fatty acid supplementation, it was concluded that the benefits of supplementation were limited with few adverse effects, but the quality of evidence across all outcomes was very low [32]. On the other hand, assessment of the improvement of fatty acid deficiency associated with omega-3 supplementation has not been the primary objective of studies, and in those in which biochemical data were included, total plasma/serum was the substrate for analysis [24,25,26] rather than specific composition of fatty acids in the phospholipid membrane of red blood cells.

As far as we are aware, results of biochemical analyses assessing a large panel of essential fatty acids in the erythrocyte membrane after long-term treatment with highly concentrated DHA in CF have not been previously reported. Therefore, the present clinical trial aimed to assess changes of n-3 PUFAs and n-6 PUFAs in the erythrocyte membrane of pediatric CF patients treated with a highly concentrated DHA product (tridocosahexaenoin 70%) or matching placebo for 12 months.

## 2. Materials and Methods

### 2.1. Design and Participants

This was a 12-month randomized, double-blind, single-center, placebo-controlled study carried out at the Pediatric Unit of Cystic Fibrosis of Parc Taulí Hospital Universitari of Sabadell, Barcelona, Spain. Eligible participants were recruited from May 2018 to February 2019 and the study finished in February 2020. The objective of the study was to determine changes in the essential fatty acid profile in the erythrocyte membrane of patients with CF treated with a highly concentrated DHA supplement for 12 months as compared with those treated with placebo.

Participants were recruited consecutively in daily practice conditions from patients already diagnosed with CF undergoing routine follow-up at our institution during the study period. Inclusion criteria were patients of both sexes, aged between 6 and 18 years old at the time of enrollment, forced expiratory volume in one second (FEV1) > 40%, and signed informed consent. Exclusion criteria were as follows: baseline oxygen saturation level < 92% or need of home supplemental oxygen therapy; history of a massive hemoptysis episode; use of systemic glucocorticoids and non-steroidal anti-inflammatory drugs within the previous 4 and 2 weeks, respectively; treatment with *CFTR* modulators; use of omega-3-containing nutritional supplements; and pregnancy or breastfeeding mothers.

The study protocol was approved by the Clinical Research Ethics Committee of Corporació Sanitària Parc Taulí (code PED-DHA-2017, approval date 18 November 2017) and was registered in the ClinicalTrials.gov (NCT04987567). Written informed consent was obtained from participants who were 18 years old and from the parents or legal guardians of participants under 17 years of age. In addition, patients between 12 and 17 years of age signed an informed assent.

### 2.2. Intervention and Study Procedures

Participants were randomized 1:1 using a permuted block design (4 or 6 subjects per block) to the experimental group (DHA supplementation) or the control group (placebo supplementation). Randomization was performed using a table of random numbers by an independent investigator from the pharmacy service of the hospital. According to the double-blind design of the study, neither the patients and their families nor the investigators (and the study personnel) were aware of the randomization allocation. 

The active product was formulated in gelatin capsules of high-rich DHA triglyceride (Tridocosahexanoin-AOX^®^ 70%). This is a concentrated DHA triglyceride, having a high antioxidant activity patented to prevent cellular oxidative damage [33,34]. The composition of the active product (Brudy NEN, BrudyLab, S.L., Barcelona, Spain) includes 400 mg of n-3 LPUFAs, 350 of which are pure DHA and 42.5 mg EPA. This supplement is registered as a food supplement for special medical purposes in the Spanish Agency for Consumer Affairs Food Safety and Nutrition (AESAN). Patients assigned to the active treatment received a dose of DHA of 50 mg/kg/day divided into one, two, or three intakes per day at the time of breakfast, lunch, and/or dinner according to the number of daily doses corresponding to the patient’s weight as follows: 2 capsules (700 mg DHA) for ≥ 13 to 17 kg, 3 capsules (1050 mg DHA) for ≥ 18 to 24 kg, 4 capsules (1400 mg DHA) for ≥ 25 to 30 kg, 5 capsules (1750 mg DHA) for ≥ 31 to 36 kg, 6 capsules (2100 mg DHA) for ≥ 37 to 43 kg, 7 capsules (2450 mg DHA) for ≥ 44 to 49 kg, and 8 capsules (2800 mg DHA) for ≥ 50 kg. The dose of DHA of 50 mg/kg (up to 2800 mg/day) was selected as this dose has been used in previous studies [26,27,28], particularly in a similar randomized, double-blind, placebo-controlled trial conducted at five CF Units in Spain [28]. Patients assigned to the control group received identically appearing gelatin capsules of olive oil and followed the same schedule. The duration of treatment was 12 months. All participants were instructed not to make any changes in their dietary habits.

Capsules returned at the end of the study were counted to determine adherence to the study treatments. Adherence was defined as consumption of at least 80% of capsules. Tolerance and product safety were assessed at the end of the study.

In order to compare the baseline fatty acid profile in the erythrocyte membrane, a group of patients without CF of the same age range (6–18 years) was included. Patients with any underlying inflammatory disease or had taken omega-3 nutraceuticals were excluded. In these patients attending the pediatric outpatient clinics of the hospital, a blood sample was obtained under routine clinical conditions and standard elective indications for laboratory testing, and in no cases were blood samples drawn as the only justification for the purpose of the study.

CF patients were also categorized according to the presence of pancreatic insufficiency as pancreatic insufficient or pancreatic sufficient, based on standard diagnosis of fecal elastase-1 (FE-1) testing. All CF patients with pancreatic insufficiency received supplemental pancreatic enzyme replacement therapy (PERT).

### 2.3. Blood Samples and Analysis of Fatty Acids

Blood samples were obtained from each participant by venipuncture in tubes with anticoagulation (EDTA or citrate) at baseline and after 6 and 12 months of treatment (end of study). After removal of plasma by centrifugation, samples were stored at −80 °C until analysis. The composition of fatty acids in red blood cells (RBC) was determined as methyl esters after a methylation reaction using the method of Lepage and Roy [35]. Samples were transferred to glass tubes for direct transesterification. Methanol-hexane 4:1 (*v*/*v*) was added with an internal standard and 0.01% butylhydroxytoluene to prevent oxidation of fatty acids. Then, acetyl chloride was added and the tubes were tightly closed with Teflon-lined caps, gasified with nitrogen, and shaken for 30 s.

Fatty acids were analyzed by gas chromatography (GC) using a gas chromatograph mass spectrometer (GCMS-QP2010 Plus, Shimadzu, Kyoto, Japan), Shimadzu AOC-20i auto injector, and Shimadzu AOC-20 autosampler. A Suprawax-280 high polarity capillary column (Teknokroma Analítica, S.A., Barcelona, Spain), 15 m × 0.10 mm internal diameter, 0.10 µm film thickness was used. Data were acquired by GCMS solution software. Functioning conditions were optimized to analyze the whole spectrum of fatty acids. Mass spectrometry operating parameters included scan rate of 10,000 amu/s, mass range of 40–400 m/z, and capillary voltage of 1.0 kV. The interface and ion source temperatures were set at 255 °C and 200 °C, respectively. The peaks of fatty acid methyl esters (FAMEs) were identified through electron ionization mass spectra using the NIST11 library and through GC retention times, comparing with a reference FAME mixture (GLC-744, Nu-Che Prep. Inc., Elysian, MN, USA). The results were expressed in relative amounts (percentage molar of total fatty acids) of duplicate sampling.

### 2.4. Panel of Fatty Acids

The panel of fatty acids analyzed included the following: saturated fatty acids (SFAs) (myristic acid C14:0, palmitic acid C16:0 [PA], stearic acid C18:0 [SA], arachidic acid C20:0, behenic acid C22:0, and lignoceric acid C24:0; monounsaturated fatty acids (MUFAs) (palmitoleic acid C16:1 n7 [POA], oleic acid C18:1 n9 [OA], cis-vaccenic acid C18:1 n7 [CVA], gondoic acid C20:1 n9, erucic acid C22:1 n9, nervonic acid (C24:1 n9), mead acid (C20:3 n9); n-6 PUFAs (linoleic acid [LA] C18:2 n6, ɣ-linolenic acid C18:3 n6, cis-11,14 eicosadienoic acid C20:2 n6, dihomo-γ-linolenic acid [DHGLA] C20:3 n6, ARA C20:4 n6, adrenic acid C22:4 n6, and osbond acid or docosapentaenoic acid [DPA n6] C22:5 n6); and n-3 PUFAs (α-linolenic acid [ALA] C18:3 n3, EPA C20:5 n3, docosapentaenoic acid [DPA n3] C22:5 n3, and DHA C22:6 n3). In addition, other fatty acid ratios were calculated, including an omega-3 index (EPA + DHA), ARA/EPA, ARA/DHA, an anti-inflammatory fatty acid index (AIFAI) ((DHGLA + EPA + DHA)/ARA), an essential fatty acid index (EFASTI) ([Σn-3 + Σn-6]/[Σn-7 + Σn-9]), n-3 PUFAs/ALA, and n-6 PUFAs/LA. On the other hand, other enzyme activity indexes were calculated, including stearoyl-CoA desaturase-18 (SDC-18) (OA/SA), delta-5-desaturase (D5D) (ARA/DHGLA), delta-6-desaturase (D6D) (DHGLA/LA), elongase 5 (C22:4 n6/ARA), and elongase 6 (SA/PA).

### 2.5. Study Outcomes

The primary outcome of the study was to assess changes in n-3 PUFAs and n-6 PUFAs in patients treated with a highly concentrated DHA supplement for 12 months. Secondary outcomes included changes SFAs, MUFAs, and fatty acid ratios.

### 2.6. Statistical Analysis

Categorical variables are expressed as frequencies and percentages and continuous variables as mean and standard deviation (SD). Differences in fatty acid composition of the erythrocyte membrane between patients with and without CF (controls) as well with and without pancreatic insufficiency were analyzed using the Student’s *t* test for independent samples. Changes of the study variables in the DHA and placebo groups during the 12-month study period as compared with baseline were assessed using the analysis of variance (ANOVA) for repeated measures with two study factors (time and study group) for paired data. The Bonferroni post-hoc multiple comparison test was used to evaluate differences between groups. Statistical significance was set at *p* < 0.05. SPSS version 25.0 (IBM Corp., Armonk, NY, USA) was used for data analysis.

## 3. Results

**Figure 1:**
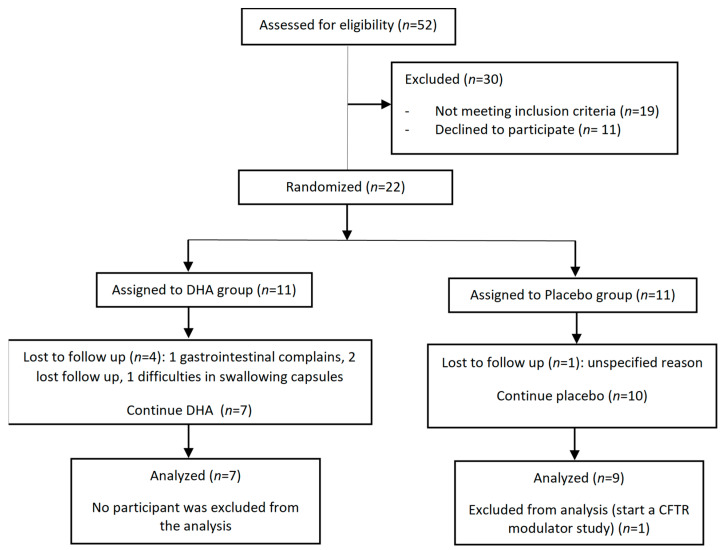
Flow chart of the study population.

During the study period, a total of 52 patients with CF were followed at the Pediatric Unit of Cystic Fibrosis of the hospital, but 30 were excluded because their ages were not in the 6–18 years range (*n* = 15), due to their current use of DHA supplementation (*n* = 4), or they declined to participate (*n* = 11). Therefore, 22 patients met the inclusions criteria and were randomized to receive DHA (*n* = 11) or placebo (*n* = 11). One patient assigned to the placebo group refused to participate and did not receive the study medication, and five patients did not complete the 12-month study period. The final analysis included seven patients in the DHA group and nine patients in the placebo group. The flow chart of the study patients is shown in Figure 1. The controls were 15 patients without CF, recruited during the study period. 

Demographic and anthropometric characteristics in patients with CF and controls were similar, and also were between the DHA and placebo groups (Table 1). Additionally, the distribution of mutations, genotype, and pancreatic-insufficient and sufficient patients between the DHA and placebo groups were similar. 

### 3.1. Baseline Fatty Acid Profile

The baseline fatty acid profile showed statistically significant differences between patients with CF and controls without CF, with higher levels of MUFAs, DHGLA, SCD-18, and D6D, and lower levels of LA, ARA, n-3 PUFAs, DHA, omega-3 index, EFASTI, and D5D in patients with CF (Table 2). Mead acid was not detected in both groups (CF patients and controls).

The baseline fatty acid profile between CF patients with pancreatic insufficiency (PI) treated with PERT and CF patients with pancreatic sufficiency (PS) showed statistically significant differences, with lower levels of SFAs, SA, n6-PUFAs, LA, EFASTI, and ARA/EPA and higher levels of MUFAs, POA, OA, ALA, and EPA; however, no statistically significant differences were observed regarding the levels of DHA, n3-PUFAs, and omega-3 index. In addition, PI patients have more SCD-18 activity and less elongase 6 (Table 3).

### 3.2. Changes of Fatty Acid Profile in the DHA and Placebo Groups

**Figure 2:**
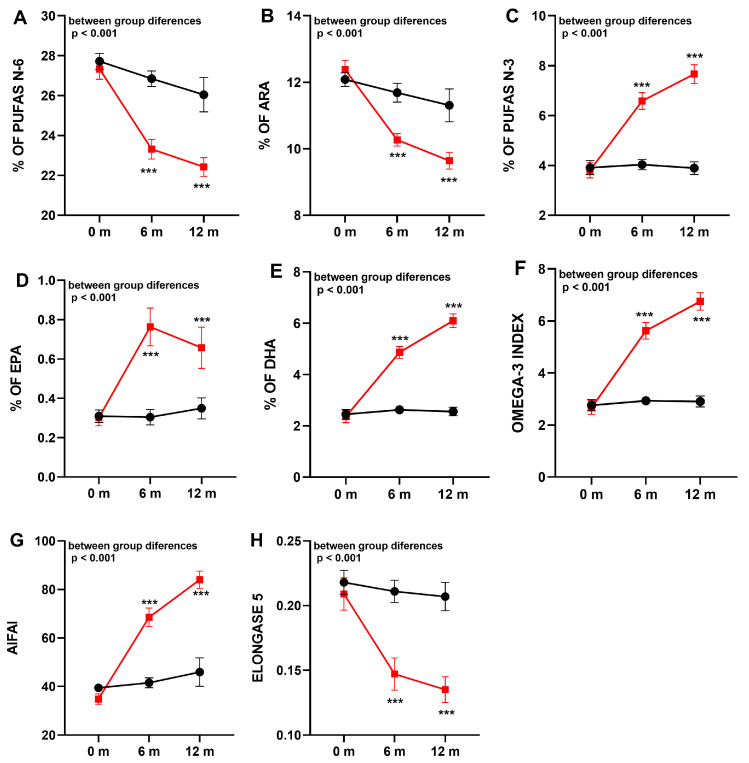
Comparison of fatty acid composition (mean ± standard error of the mean, SEM) in the erythrocyte membrane in the groups of supplementation with DHA (squares, red line) or placebo (circles, black line). (**A**): n-6 polyunsaturated fatty acids (PUFAs); (**B**): arachidonic acid (ARA); (**C**): n-3 PUFAs; (**D**): eicosapentaenoic acid (EPA); (**E**): docosahexaenoic acid (DHA); (**F**): omega-3 index; (**G**): anti-inflammatory fatty acid index (AIFAI); (**H**): elongase 5 activity. *** *p* < 0.001 indicating statistically differences into groups.

The results of fatty acid profiles in the two study groups are shown in Table 4 and Figure 2. In the placebo group, the profile of fatty acids remained stable over the 12-month study period. However, in patients receiving the DHA supplement, there was a statistically significant improvement of n-3 PUFAs, which was already observed at 6 months and further increasing at 12 months (from 3.95% at baseline to 7.67% at 12 months, *p* < 0.001). Among n-3 PUFAs, there were significant increases in DHA (from 2.48% at baseline to 6.10% at 12 months, *p* < 0.001) and EPA (from 0.33% at baseline to 0.66% at 12 months, *p* < 0.001), which were associated with a significant increase in the omega-3 index. In contrast to increasing values of n-3 PUFAs, a statistically significant decrease of n-6 PUFAs was found (from 27.21% at baseline to 22,43% at 12 months, *p* = 0.009), mostly at the expense of a decrease in ARA levels (from 12.42% at baseline to 9.64% at 12 months, *p* < 0.001). Changes in LA levels were not observed. These observed changes were translated into a statistically significant improvement in inflammatory markers (ARA/EPA, ARA/DHA, and AIFAI) in the supplemented patients with respect to the placebo group. Finally, DHA administration only reduces elongase 5 enzyme activity.

### 3.3. Safety and Adherence

Of all patients included in the study, one patient treated with DHA reported intermittent abdominal pain and *Giardia lamblia* was detected in a stool sample. The patient was successfully treated with metronidazole, but the parents requested withdrawal of the study. The relationship with the study medication could not be established. No other potential adverse events were registered. The mean percentage of adherence was 85.8% without statistically significant differences between the DHA (92.4%) and placebo (77.5%) groups (*p* = 0.071).

## 4. Discussion

The present study carried out in a sample of pediatric patients with CF confirms alterations of essential fatty acids already described in CF [4,13,14]. At baseline and compared with normal controls, CF patients showed significantly lower levels of n-3 PUFAs (at the expense of DHA) and n-6 PUFAs including LA and ARA, as well as an increase of MUFAs, without changes in SFAs. In our study, mead acid was not detected in both groups of CF patients and controls. This may be due to the fact that we have analyzed it in the erythrocyte membrane and the other studies have evaluated mead acid in plasma or serum [9,36]. 

Fatty acid dysregulations in CF cells have been explained by the interaction of different mechanisms, such as malabsorption of precursor due to exocrine pancreatic insufficiency, fibrosis-related liver disease, hypoxia affecting fatty acid desaturases, increased flux through the n-6 omega pathway, release of ARA from phospholipids by phospholipase A2, and alterations of membrane biophysical properties [37]. In our study, we observed that this fatty acid dysregulation may be due to statistically significant differences in desaturase activities (SCD-18, D5D and D6D) between CF group and controls, as observed by Lloyd-Still et al. [17].

We also observed that CF patients with pancreatic insufficiency, despite receiving PERT, present higher levels of OA and lower levels of LA, without statistically significant differences in ARA, as also found Habbard et al. [11] and Freedman et al. [6]. The recovery of intestinal absorption by the addition of pancreatic enzymes does not assure the same fatty acid composition in CF patients. In this sense, we observed a decrease in SA levels and an increase in OA levels related to the higher SCD-18 activity in IP patients. In addition, the levels of n-6 PUFAs, especially LA, and EFASTI are decreased and the levels of ALA and EPA are increased in pancreatic-insufficient patients compared to pancreatic-sufficient patients. However, there were no differences in DHA levels. These results reinforce the hypothesis that fatty acid metabolism is correlated with the degree of dysfunction of the *CFTR* protein, as CF patients with pancreatic insufficiency have more severe mutations than those without insufficiency, supporting the results of Freedman et al. [6] who showed that carriers tend to have intermediate fatty acid levels between patients with CF and controls.

In relation to the characteristics of the study population, the mean age was 11.7 years (range 6–12), which is lower than that reported in most studies of fatty acid profiles in CF patients focused on adult populations or mixed children and adults [24,30,31,38], but similar to studies in pediatric patients [27,29]. Although there were more males in the DHA group than among patients treated with placebo, this difference did not influence the baseline fatty acid profiles in both study groups.

Long-term supplementation with a highly concentrated re-esterified triglyceride of DHA (tridocosahexaenoin-AOX^®^ 70%) from fish oil of high bioavailability, at doses of 50 mg/kg/day, was associated with very rapid and significant increase of DHA in phospholipids of the erythrocyte membrane as compared with placebo, both at 6 (2.08-fold increase) and 12 months (3.62-fold increase) of treatment. Additionally, a significant increase in EPA levels was also observed. Supplementation with this DHA product that incorporates around 10% of EPA (each capsule of 400 mg of omega-3 included 350 mg of DHA and 42.5 mg of EPA) is sufficient to achieve optimal levels of EPA. However, oxidation of DHA by the peroxisomes may contribute to EPA via the retroconversion pathway [38]. The omega-3 index also increased significantly as a result of increases in DHA and EPA. Patients treated with DHA, as compared with placebo, not only showed an increase in n-3 PUFAs in the erythrocyte membrane, but also a statistically significant reduction of n-6 PUFAs at the expense of a significant decrease in ARA. Increases in DHA and EPA levels and decreases in ARA accounted for lower ARA/DHA and ARA/EPA ratios, lower elongase 5 activity, and higher AIFAI at the end of study. Although the placebo capsules contained small quantities of olive oil, levels of SFAs, MUFAs, and PUFAs in the erythrocyte membrane remained unchanged throughout the study period. In addition, LA and EFASTI levels are also unchanged.

In agreement with the present findings, other randomized controlled trials in pediatric patients with CF have shown an increase in blood and tissue DHA levels after DHA algal triacylglycerol oil (50 mg/kg/day) for 6 months [27], decreases in ARA/DHA ratio with high-dose DHA (100 mg/kg/day in the first month followed by 1 g/day for 12 months) [30], and significant increase in serum DHA and EPA concentrations with a concomitant decrease in ARA was observed after 1-year supplementation with algal-rich DHA oil [39]. In none of these previous trials, however, has a complete panel of fatty acid profiles in the erythrocyte membrane after long-term DHA supplementation been reported.

The relevance of assessing fatty acids in the erythrocyte phospholipid membrane vs. total plasma/serum fatty acid profiling merits a comment. It is important to note that when measuring the lipid composition of blood, various blood compartments have been targeted [40]. Plasma or serum fatty acid levels have been extensively examined as they reflect short-term dietary fat intake. However, analyzing lipid compositions from mature red blood cell membranes provides an advantage over plasma analysis because red blood cells last an average of 120 days in the blood, compared to 3 weeks for platelet or plasma lipids. This longer lifespan reflects better representation of long-term dietary fatty acid intake and tissue conditions [41]. Furthermore, red blood cell membranes maintain a more stable fatty acid composition compared to plasma fatty acid levels [42]. In fact, monitoring the individual’s erythrocyte membrane fatty acid profile can serve as an excellent biomarker for personalized dietary interventions aimed at addressing fatty acid deficiencies and preventing or controlling diseases. By tracking the optimal intake, membrane incorporation, and biochemical transformations, this approach allows for a tailored approach to dietary intervention. Fatty acids in phospholipids are influenced by both nutritional and metabolic factors, with a significant contribution from the individual’s metabolism and the overall health status of the patients.

The reduced sample size is the main limitation of the study, which is related to the single-center nature of the study. Despite the strong recommendation to maintain dietary habits throughout the period of supplementation, dietary intake during the study was a variable that was not strictly controlled.

## 5. Conclusions

The present findings indicate that the recovery of intestinal absorption by adding pancreatic enzymes does not guarantee the same basal fatty acid composition in pediatric CF patients under 18 years of age. However, imbalances in n-6 and n-3 long-chain PUFAs, as well as the ARA/DHA ratio and inflammatory markers associated with fatty acids, can be normalized by using a high-rich DHA supplement (tridocosahexaenoin-AOX^®^ 70%) at a dose of 50 mg/kg/day. However, it is important to note that essential fatty acid alterations cannot be fully normalized with this dietary supplementation. Long-term administration of a highly concentrated DHA for over one year has been shown to be safe and well tolerated. A comprehensive characterization of all essential fatty acid families and the main ratios in erythrocyte membranes contributes to a better knowledge of fatty acid deficiencies in pediatric patients with CF. This information is also valuable for comparison purposes in future research studies. 

## Figures and Tables

**Table 1:** Demographic, anthropometric, and clinical characteristics of the study population.

Variables	Patients with Cystic Fibrosis (*n* = 22)		Controls (*n* = 15)	*p* Value
	DHA Group (*n* = 11)	Placebo Group (*n* = 11)		
Gender, male, *n* (%)	7 (63.6)	3 (23.3)	9 (60)	0.160
Age, years, mean (SD)	10.9 (3.2)	12.5 (3.8)	10.9 (2.7)	0.381
Weight, kg, mean (SD)	39.7 (17.4)	43.4 (14.6)	41.5 (17.5)	0.803
Height, cm, mean (SD)	145.2 (20.2)	149.3 (19.0)	143.6 (17.2)	0.743
Body mass index, kg/m^2^, mean (SD)	17.9 (2.9)	19.3 (2.4)	19.3 (3.8)	0.501
Mutations, *n* (%) *				
Severe/severe	7 (63.6)	5 (45.5)		0.668
Severe/mild	3 (27.3)	4 (36.4)		1.0
Mild/mild	1 (9.1)	2 (18.2)		1.0
Genotype, *n* (%)				
ΔF508/ΔF508	3 (27.3)	1 (9.1)		0.580
ΔF508del/other	5 (45.5)	7 (63.6)		0.668
Other/other	3 (27.3)	3 (27.3)		1.0
Pancreatic insufficiency, *n* (%)	7 (63.6)	5 (45.5)		0.668
Chronic *S. aureus* infection, *n* (%)	3 (27.3)	2 (18.2)		1.0
Chronic *P. aeruginosa* infection, *n* (%)	0	1 (9.1)		1.0
FEV_1_, %, mean (SD)	92.36 (9.8)	97.39 (15.5)		0.373

* Severe mutations: mutations with mild or minimal function of *CFTR* (group I, II or III mutations); mild mutations: mutations with residua *CFTR* function (group IV, V, VI or VII mutation); SD: standard deviation.

**Table 2:** Baseline fatty acid profile in the erythrocyte membrane of patients with CF and controls.

Fatty Acids	Patients with CF (*n* = 22)	Controls (*n* = 15)	*p* Value
SFAs, %	46.78 (1.03)	47.04 (0.5)	0.380
PA	20.63 (0.63)	20.80 (0.80)	0.477
SA	17.85 (0.82)	17.68 (0.55)	0.395
MUFAs, %	21.82 (1.8)	19.90 (1.2)	**0.001**
POA	0.28 (0.11)	0.22 (0.04)	0.112
CVA	1.65 (0.28)	1.46 (0.15)	**0.024**
OA	14.03 (1.09)	12.87 (0.86)	**0.002**
n-6 PUFAs, %	27.52 (1.3)	28.51 (1.7)	0.051
LA	10.01 (0.85)	10.64 (0.89)	**0.042**
DHGLA	1.81 (0.35)	1.44 (0.25)	**0.002**
ARA	12.23 (0.67)	12.79 (0.74)	**0.023**
n-3 PUFAs, %	3.86 (0.8)	4.54 (1.1)	**0.033**
ALA	0.08 (0.02)	0.07 (0.02)	0.797
EPA	0.31 (0.09)	0.37 (0.20)	0.232
DHA	2.31 (0.02)	3.01 (0.8)	**0.002**
Omega-3 index	2.71 (0.6)	3.38 (0.9)	**0.013**
EFASTI	1.45 (0.2)	1.67 (0.15)	**<0.001**
ARA/EPA	45.1 (16.3)	48.33 (29.5)	0.67
ARA/DHA	5.42 (1.5)	5.02 (0.64)	0.26
AIFAI	0.37 (0.1)	0.38 (0.1)	0.715
n-3 PUFAs/LA	51.46 (23.3)	64.90 (23.5)	0.095
n-6 PUFAs/LA	1.75 (0.2)	1.69 (0.2)	0.267
SCD-18	0.79 (0.08)	0.72 (0.07)	**0.011**
D5D	7.04 (1.60)	9.09 (1.41)	**0.003**
D6D	0.18 (0.04)	0.14 (0.03)	**<0.001**
Elongase 5	0.19 (0.08)	0.21 (0.003)	0.228
Elongase 6	0.85 (0.04)	0.87 (0.05)	0.278

Data as mean (standard deviation, SD). SFAs: saturated fatty acids, PA: palmitic acid, SA: stearic acid, MUFAs: monounsaturated fatty acids, POA: palmitoleic acid, CVA: cis-vaccenic acid, OA: oleic acid, PUFAs: polyunsaturated fatty acids; LA: linoleic acid, DHGLA: dihomo-γ-linolenic acid, ARA: arachidonic acid, ALA: α-linolenic acid, EPA: eicosapentaenoic acid, DHA: docosahexaenoic acid, EFASTI: essential fatty acid index, AIFAI: anti-inflammatory fatty acid index, SCD-18: stearoyl-CoA desaturase-18, D5D: delta-5-destaturase, D6D: delta-6-desaturase. Bold values denote statistically significance at the *p* < 0.05.

**Table 3:** Differences between CF patients with and without pancreatic insufficiency.

Fatty Acids	Pancreatic Sufficient CF Patients (*n* = 10)	Pancreatic Insufficient CF Patients (*n* = 12)	*p* Value
SFAs, %	47.42 (0.63)	46.26 (1.03)	**0.009**
PA	20.62 (0.66)	20.96 (0.91)	0.282
SA	18.09 (0.48)	17.33 (0.34)	**<0.001**
MUFAs, %	20.76 (1.15)	22.72 (1.72)	**0.017**
POA	0.22 (0.04)	0.33 (0.12)	**0.048**
CVA	1.60 (0.28)	1.70 (0.29)	0.496
OA	13.40 (0.84)	14.56 (1.0)	**0.014**
n-6 PUFAs, %	28.22 (0.97)	26.95 (1.23)	**0.020**
LA	10.59 (0.71)	9.55 (0.67)	**0.004**
DHGLA	1.75 (0.41)	1.86 (0.31)	0.418
ARA	12.32 (0.70)	12.16 (0.66)	0.355
n-3 PUFAs, %	3.60 (0.88)	4.07 (0.70)	0.159
ALA	0.064 (0.014)	0.089 (0.027)	**0.009**
EPA	0.26 (0.10)	0.34 (0.08)	**0.014**
DHA	2.31 (0.56)	2.49 (0.62)	0.381
Omega-3 index	2.57 (0.63)	2.83 (0.66)	0.346
EFASTI	1.54 (0.12)	1.37 (0.14)	**0.020**
ARA/EPA	53.49 (17.47)	37.29 (9.85)	**0.020**
ARA/DHA	5.58 (1.21)	5.23 (1.62)	0.417
AIFAI	0.35 (0.04)	0.39 (0.06)	0.159
n-3 PUFAs/LA	60.12 (27.67)	47.53 (14.43)	0.346
n-6 PUFAs/LA	1.67 (0.14)	1.82 (0.18)	**0.042**
SCD-18	0.74 (0.06)	0.84 (0.06)	**0.003**
D5D	7.43 (1.95)	6.72 (1.22)	0.418
D6D	0.16 (0.04)	0.19 (0.03)	0.072
Elongase 5	0.22 (0.03)	0.21 (0.03)	0.321
Elongase 6	0.88 (0.03)	0.83 (0.04)	**0.006**

Data as mean (standard deviation, SD). SFAs: saturated fatty acids, PA: palmitic acid, SA: stearic acid, MUFAs: monounsaturated fatty acids, POA: palmitoleic acid, CVA: cis-vaccenic acid, OA: oleic acid, PUFAs: polyunsaturated fatty acids; LA: linoleic acid, DHGLA: dihomo-γ-linolenic acid, ARA: arachidonic acid, ALA: α-linolenic acid, EPA: eicosapentaenoic acid, DHA: docosahexaenoic acid, EFASTI: essential fatty acid index, AIFAI: anti-inflammatory fatty acid index, SCD-18: stearoyl-CoA desaturase-18, D5D: delta-5-destaturase, D6D: delta-6-desaturase. Bold values denote statistically significance at the *p* < 0.05.

**Table 4:** Fatty acid profile in the erythrocyte membrane in the DHA and placebo groups during the 12-month study period.

Fatty Acids	Placebo Group			DHA Group			*p* Value		
	Baseline (*n* = 9)	6 Months (*n* = 9)	12 Months (*n* = 9)	Baseline (*n* = 7)	6 Months (*n* = 7)	12 Months (*n* = 7)	Time *	Treatment ^†^	Interaction ^‡^
SFAs, %	47.19 (0.55)	47.23 (0.85)	48.51 (2.14)	46.20 (1.09)	48.42 (1.53)	48.23 (1.26)	**0.012**	0.672	0.237
PA	20.87 (0.74)	20.98 (0.83)	22.06 (1.76)	20.74 (0.90)	21.92 (0.93)	22.56 (0.61)	**0.001**	0.164	0.364
SA	17.76 (0.41)	17.82 (0.63)	17.99 (0.43)	17.59 (0.68)	18.01 (0.70)	17.36 (0.61)	0.419	0.239	0.151
MUFAs, %	21.08 (1.23)	21.80 (0.99)	21.70 (1.19)	22.63 (1.87)	21.53 (2.42)	21.67 (1.95)	0.550	0.898	0.995
POA	0.29 (0.11)	0.27 (0.06)	0.28 (0.07)	0.26 (0.11)	0.22 (0.07)	0.25 (0.08)	0.599	0.135	0.878
CVA	1.58 (0.2)	1.51 (0.25)	1.37 (0.15)	1.74 (0.34)	1.41 (0.18)	1.34 (0.15)	**0.001**	0.876	0.239
OA	13.79 (0.86)	14.11 (0.83)	14.18 (1.07)	14.28 (1.28)	13.89 (2.16)	14.32 (1.1)	0.785	0.592	0.327
n-6 PUFAs, %	27.86 (1.27)	27.00 (1.10)	25.95 (2.71)	27.21 (1.34)	23.33 (1.41)	22.42 (1.23)	**<0.0001**	**<0.0001**	**0.009**
LA	10.17 (0.86)	9.98 (0.80)	9.56 (1.23)	9.60 (0.82)	9.63 (0.93)	9.45 (0.67)	0.349	0.569	0.980
DHGLA	1.99 (0.36)	1.88 (0.41)	1.84 (0.57)	1.63 (0.26)	1.39 (0.12)	1.33 (0.11)	0.269	**<0.0001**	0.116
ARA	12.14 (0.67)	11.79 (0.82)	11.35 (0.51)	12.42 (0.77)	10.20 (0.49)	9.64 (0.66)	**0.001**	**<0.0001**	**0.007**
n-3 PUFAs, %	3.85 (0.87)	3.96 (0.61)	3.83 (0.78)	3.95 (0.83)	6.71 (0.88)	7.67 (0.99)	**<0.001**	**<0.001**	**<0.001**
ALA	0.07 (0.02)	0.08 (0.04)	0.09 (0.02)	0.08 (0.03)	0.09 (0.02)	0.08 (0.03)	0.673	0.768	0.416
EPA	0.29 (097)	0.27 (0.84)	0.33 (0.16)	0.33 (0.10)	0.80 (0.24)	0.66 (0.47)	**0.001**	**<0.0001**	**0.002**
DHA	2.31 (0.01)	2.61 (0.44)	2.51 (0.51)	2.48 (0.46)	4.94 (0.59)	6.10 (0.72)	**<0.0001**	**<0.0001**	**<0.0001**
Omega-3 index	2.69 (0.66)	2.89 (0.49)	2.85 (0.65)	2.73 (0.76)	5.75 (0.82)	6.75 (0.88)	**<0.0001**	**<0.0001**	**<0.0001**
ARA/EPA	45.80 (18.05)	46.49 (14.17)	43.56 (23.53)	41.18 (14.03)	13.91 (4.77)	17.68 (9.11)	**0.011**	**0.0008**	**0.017**
ARA/DHA	5.38 (1.37)	4.64 (0.86)	4.75 (1.11)	5.64 (1.87)	2.10 (0.31)	1.6 (0.18)	**<0.0001**	**<0.0001**	**<0.0001**
AIFAI	0.39 (0.04)	0.41 (0.06)	0.41 (0.10)	0.36 (0.06)	0.70 (0.09)	0.84 (0.09)	**<0.0001**	**<0.0001**	**<0.0001**
EFASTI	1.51 (0.13)	1.42 (0.09)	1.38 (0.16)	1.39 (0.16)	1.47 (0.34)	1.40 (0.17)	0.558	0.552	0.692
n-3 PUFAs/ALA	61.44 (31.17)	50.27 (13.7)	43.12 (12.45)	43.43 (50.28)	79.55 (13.65)	97.45 (34.72)	**0.022**	**0.0006**	**0.0003**
n-6 PUFAs/LA	1.74 (0.18)	1.71 (0.16)	1.70 (0.15)	1.85 (0.15)	1.42 (0.09)	1.37 (0.10)	**0.002**	**<0.0001**	**0.003**
SCD-18	0.78 (0.05)	0.79 (0.05)	0.79 (0.07)	0.81 (0.09)	0.77 (0.13)	0.082 (0.07)	0.719	0.448	0.543
D5D	6.30 (1.40)	6.58 (1.99)	6.73 (3.31)	7.79 (1.47)	7.48 (0.96)	7.29 (0.63)	0.999	0.064	0.761
D6D	0.20 (0.04)	0.19 (0.03)	0.18 (0.04)	0.17 (0.03)	0.14 (0.02)	0.14 (0.02)	0.167	**0.0006**	0.899
Elongase 5	0.22 (0.03)	0.21 (0.03)	0.21 (0.03)	0.21 (0.03)	0.14 (0.03)	0.15 (0.03)	**0.0006**	**<0.0001**	**0.010**
Elongase 6	0.85 (0.04)	0.85 (0.05)	0.82 (0.05)	0.85 (0.05)	0.82 (0.05)	0.77 (0.03)	**0.005**	0.064	0.388

Data as mean (standard deviation, SD); * Between-group comparison; ^†^ Between-time comparison; ^‡^ Interaction between group and time. Bold values denote statistically significance at the *p* < 0.05. SFAs: saturated fatty acids, PA: palmitic acid, SA: stearic acid, MUFAs: monounsaturated fatty acids, POA: palmitoleic acid, CVA: cis-vaccenic acid, OA: oleic acid, PUFAs: polyunsaturated fatty acids, LA: linoleic acid, DHGLA: dihomo-γ-linolenic acid, ARA: arachidonic acid, ALA: α-linolenic acid, EPA: eicosapentaenoic acid, DHA: docosahexaenoic acid, EFASTI: essential fatty acid index, AIFAI: anti-inflammatory fatty acid index, SCD-18: stearoyl-CoA desaturase-18, D5D: delta-5-destaturase, D6D: delta-6-desaturase.

## Data Availability

Data are available from the authors (R.A-V. and J.C.D.) upon request.
